# Editorial: Adaptive response of living beings to extreme environments: Integrative approaches from cellular and molecular biology, biotechnology, microbiology to physiology

**DOI:** 10.3389/fphys.2022.1068287

**Published:** 2022-11-03

**Authors:** David Cristobal Andrade, Benito Gómez-Silva, Ramon Alberto Batista-García, Gregoire P. Millet

**Affiliations:** ^1^ Exercise Applied Physiology Laboratory, Centro de Investigación en Fisiología y Medicina de Altura, Departamento Biomedico, Facultad de Ciencias de La Salud, Universidad de Antofagasta, Antofagasta, Chile; ^2^ Laboratory of Biochemistry, Biomedical Department and Centre for Biotechnology and Bioengineering (CeBiB), Universidad de Antofagasta, Antofagasta, Chile; ^3^ Centro de Investigación en Dinámica Celular, Instituto de Investigación en Ciencias Básicas y Aplicadas, Universidad Autónoma Del Estado de Morelos, Cuernavaca, Mexico; ^4^ Institute of Sport Sciences, University of Lausanne, Lausanne, Switzerland

**Keywords:** extreme environments, high altitude, exercise physiology, inhospital, diving

## Introduction

For billions of years, since our first ancestors appeared, natural environmental conditions have been a driving force in the evolution of life on our planet. Adaptive mechanisms shown by successful unicellular or multicellular organisms in extreme environments are essential to their survival and reproduction. Natural conditions, such as high altitude, high gravity, extreme pressures, radiation, cold, heat, salinity, oligotrophic, and others, are examples of stressful factors that challenge the physical and chemical limits at which life can persevere. Among higher organisms, physiological manifestations such as cardiorespiratory adjustments for oxygen homeostasis are adaptive strategies allowing humans to work and live at high altitudes (Beltran et al., 2020; Mallet et al., 2021; Lang et al.). Along with this, some human activities are performed in severe environmental conditions, such as diving, and races during extreme heat and cold, among others, which demonstrate the human capacity to push their limits to be able to respond and adapt to conditions where human life would be a great challenge (Arce-Alvarez, et al., 2021; Arce-Alvarez, et al., 2022). Also, other adaptive responses relative to the photosynthetic process have been reported in plants from drylands. In microorganisms inhabiting extreme environments such as hot and cold hyperarid deserts, extensive research has been reported on both diversity and abundance, as well as metabolic pathways, gene products, and bioactive evaluation of promising biomolecules (Bull et al., 2016; Gómez-Silva, 2018; Gómez-Silva and Batista-Garcia, 2022). Receptor overexpression, second messengers, and/or secondary metabolites are some examples relative to adaptive processes promoted by one or more extreme environmental conditions, which could even be used in biomedicine, demonstrating the translational potential of human physiology.

Despite technological advances, adaptation to extreme environments remains a challenge to research on cellular and molecular biology, biochemistry, biotechnology, microbiology, and physiology. Indeed, one example is habitability at high-altitude with low temperatures and high UV irradiation that are colonized by psychrophilic organisms (mainly bacteria and archaea), which has been adapted to growth in a wide temperature range (−15°C–20°C) and under hypobaric conditions. Also, research on the effects and adaptation to intensive mining activity in high-altitude regions (e.g., Australia, Russia, Brazil, and Chile) is increasingly investigated (Lang et al.). Thus, research in inhospitable environments, apparently devoid of life forms, has shown the resilience of life on our planet.

This special issue was focused on cellular and molecular biology and physiological adaptive mechanisms at the cell level but also at tissue and organ levels. Accordingly, this Research Topic issue emphasizes the adaptive strategies in environmental conditions involving humans, animals, cell models, and microorganisms, particularly considering physiological and cellular adaptive mechanisms at the cellular/tissue/organ level. Thus, considering the interest in the evidence presented in our topic, the readers can briefly overview the multitude of topics and the main findings reported in the included articles.

### High altitude: A human health challenge

Several human activities are performed at high altitudes, such as tourism, sports, and mining. In fact, in Los Andes mountains, many mining industries are located, which carry out their work activities in high-altitude hypoxic environments. In our Research Topic, Lang et al. showed that in hypertensive miners (*n* = 10) high altitude was the main factor affecting cardiac autonomic modulation, independently of hypertension. Of note, apparently, the parasympathetic arm of the autonomic nervous system was more affected by high altitude, similar to previous observations (Beltran et al., 2020). Importantly, Lang et al. also showed that hypertension was associated with reduced physical performance independently from altitude reached with a higher respiratory frequency. In addition, it has been proposed that iron metabolism could play a pivotal role in adaptations to high altitudes, favoring oxygen transport. Accordingly, in a perspective manuscript, Zhang Y et al. proposed that high fluoride levels can reduce the antioxidant capacity while increasing the contents of reactive oxygen species and malondialdehyde, resulting in oxidative stress and fluoride-induced oxidative stress, which are related to iron metabolism disorders.

Acute mountain sickness is hypoxia-induced severe symptoms such as headache, fatigue, and insomnia and has been recognized as one of the more fundamental problems related to high altitude exposure. Searching for possible solutions or palliatives, Rupp et al. proposed and showed that positive expiratory pressure could be an effective maneuver to acute mountain sickness in trakkers. Indeed, they showed that at 5,085 m, 10 cmH_2_O breathing improved arterial and tissue oxygenation, as well as symptoms of acute mountain sickness after trekking to a very high altitude (*n* = 24), although reduced cerebral perfusion and cardiac output. Along with this, Zhang J et al. showed that YQ23, a novel bovine-derived with an excellent capacity for carrying and releasing oxygen, could be used to reduce acute mountain sickness in preclinical models. The authors achieve to prove that YQ23 effectively alleviates acute altitude hypoxia injury of acute mountain sickness with no adverse effects on hemodynamics, myocardial ischemia, and renal injury (Zhang J et al.).

### Diving activities and cold: Other forms of extreme environment

Extreme environments are not only closely related to high altitude; if not, they could be associated with any internal or external human challenge; and diving activities could be considered one of these. In fact, elevated ambient pressure, immersion, cold, hyperoxia, and/or changes in breathing-gas features could affect activities such as diving. It is known that helium is less soluble and more diffusive than nitrogen; nevertheless, helium-oxygen (heliox) mixtures have been used as a breathing medium during deep dives to avoid the narcotic effects of nitrogen under pressure. However, although heliox has been commonly used, the possible impact on physiological function is not completely describe. Accordingly, in the current Research Topic, Bao et al. tested whether single deep heliox dive affected the physiological function of 40 male divers who performed an open-water heliox dive to 80 m of seawater. They showed that heliox dive activates the endothelium, causes skeletal muscle damage, and induces oxidative stress and physiological stress responses (biomarkers).

In addition to physiological function, diving activities could affect all life forms, including the bacteria that reside on human skin and mucosa. Accordingly, Monnoyer et al. examined the metabolic activity of oral bacteria from healthy males (*n* = 23) during commercial heliox saturation diving through pathway abundance analysis using PICRUSt2, a bioinformatics software package that uses marker gene data to compute the metabolic activity of microbial communities. The authors showed that predicted changes were transient and appeared primarily associated with the survival and growth of bacteria in hyperoxic environments, as well as a reduction in bacterial vitB_12_ biosynthesis.

In addition to hypoxic environments and diving activities, the cold could be considered within the characteristics of inhospitable places. Pigs are susceptible to low-temperature conditions, and cold stress causes metabolic changes in the body to counteract cold. Accordingly, Chen et al. subjected sixteen 30-day-old male weaned piglets at different cold exposure times, and liver samples were analyzed using systemic non-targeted metabolomics to determine metabolic changes induced by cold. The authors showed that most pathways were related to amino acid or energy metabolism. Notably, 2 and 6 h of cold exposure were more related to carbohydrates and energy production metabolic pathways, and the following 12 h of cold exposure were more related to the metabolism connected with epinephrine (autonomic functions) (Chen et al.).
